# Superior vena cava syndrome in a patient with previous cardiac surgery: what else should we suspect?

**DOI:** 10.1186/1746-1596-5-43

**Published:** 2010-06-25

**Authors:** Panagiotis Dedeilias, Ioannis Nenekidis, Panagiotis Hountis, Christos Prokakis, Paraskevi Dolou, Efstratios Apostolakis, Efstratios N Koletsis

**Affiliations:** 1 1st Cardiac Surgery Department, "Evangelismos" General Hospital, Athens, Greece; 2 Department of Cardiothoracic Surgery, University of Patras, Greece; 3 Department of Anaesthesiology, Evangelismos General Hospital, Athens, Greece

## Abstract

**Background:**

Although mediastinal tumors compressing or invading the superior vena cava represent the major causes of the superior vena cava syndrome, benign processes may also be involved in the pathogenesis of this medical emergency. One of the rarest benign causes is a pseudoaneurysm developing in patients previously having heart surgery.

**Case report:**

We present the case of a large pseudoaneurysm of the ascending aorta, five years after primary surgery, with a significant compression of the right mediastinal venous system causing superior vena cava syndrome, detected at chest CT angiography. Perioperative findings showed two rush out points both coming from the distal aortic suture line which was performed five years ago. The patient underwent reoperation under circulatory arrest facilitating safe exploration and repair of the distal anastomotic leaks

**Conclusion:**

Enhanced chest CT should be always undertaken in all patients with superior vena cava syndrome, especially in those previously having cardiac or aortic surgery to correctly evaluate the presence of a pseudoaneurysm. Mass effect to the superior vena cava makes necessary an open surgical treatment of the pseudoaneurysm so as to concurrently resolve the right mediastinal venous system's compression. Surgery should be performed in terms of safe approach to avoid exsanguination and cerebral malperfusion.

## Background

The superior vena cava syndrome is mostly related to the compression or invasion of the vessel by mediastinal tumors. Among the few benign causes still reported in the literature, the pseudoaneurysm of the ascending aorta is the rarest. A -propos of a case report, we briefly present the current knowledge on the evaluation and management of patients presenting with mediastinal enlargement and superior vena cava syndrome due to an ascending aorta pseudoaneurysm.

## Case Report

A 79 year old man was admitted at our emergency department due to exertion dyspnoea and swelling of the upper limbs, head and neck. Five years ago the patient had undergone elective replacement of the aortic valve with a prosthetic one (Carbomedics 23 mm) and a concomitant ascending aorta aneurysm replacement with a 28 mm synthetic graft. On admission the patient was dyspnoic with malaise. His blood pressure was normal (128/82 mmHg) and the oxygen saturation without oxygen supply was 92%. Pulses were irregular and increased in rate. ECG showed atrial fibrillation with fast ventricular response (135 to 145 beats/min). The physical examination documented cyanotic and swollen head and neck and distended jugular veins. Superior vena cava syndrome (SVCS) was clinically diagnosed. Laboratory data included a prolonged INR of 7 and Ht: 35.6%. Other elements of blood count and coagulation time were within physiologic values. Cardiac enzymes were negative. LDH was 620 IU/L. The rest of his biochemical profile was normal. Arterial blood gases showed mild respiratory acidosis (PH: 7.33) due to elevated PCO_2_: 48mmHg.

**Figure 1:**
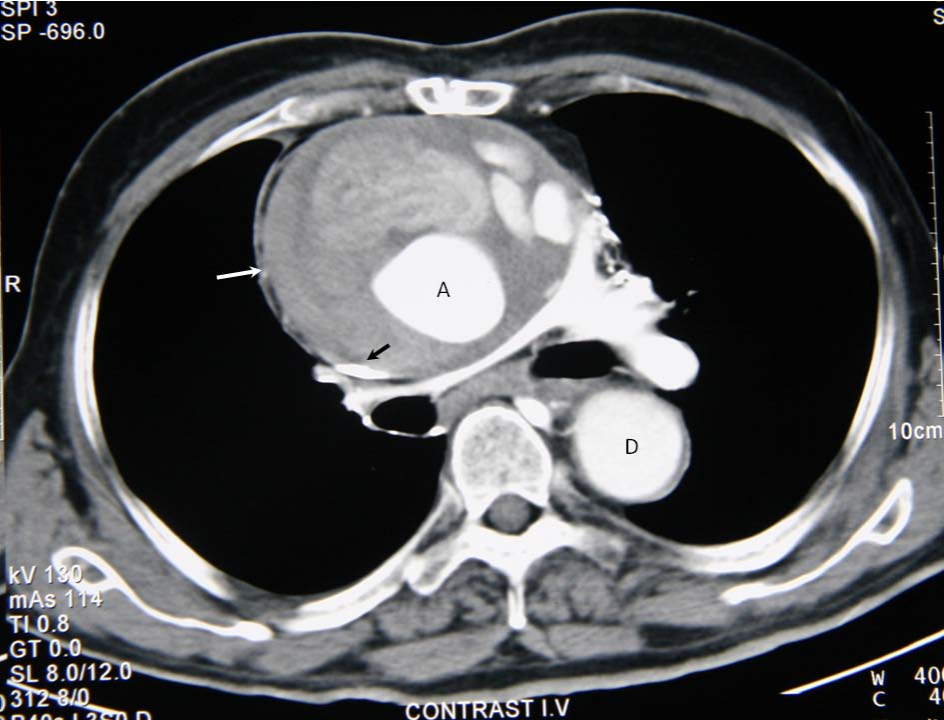
**Contrast CT scan**. Contrast computed tomography of the superior mediastinum showing (white arrow) the pseudoaneurysm of the aorta causing compression of superior vena cava (black arrow). (A denotes ascending aorta, D denotes descending aorta).

Chest x-rays was further confirmatory of the clinical diagnosis showing a widened mediastinum. However the contrast CT angiography of the chest was remarkable, revealing a large pseudoaneurysm of the ascending aorta with a maximum diameter of 13cm compressing the superior vena cava and an extensive collateral circulation (**Figure **1).

Transfusions with 2 units of fresh frozen plasma were enough to lower the INR to 1.8 and the patient was then transferred to the operating room. Femoro-femoral cannulation was undertaken in order to establish cardiopulmonary bypass and decrease the patient's temperature to down to 16°C to safely commence circulatory arrest. After the completion of cooling with collection of the patient's blood within the reservoir of the extracorporeal device, repeat sternotomy was performed through safe surgical means. Retrograde cardioplegia was installed. The pseudoaneurysm was incised and the thrombotic material was carefully removed. Two sites of major leakage originating from the anterior and posterior segment of the distal anastomosis were revealed. Suturing with 3/0 prolene, reinforced by teflon patches, was performed. Eventually extracorporeal circulation was recommenced, gradual rewarming was carried out and successful weaning from cardiopulmonary bypass was achieved.

The patient was extubated 6 hours later and he remained in the intensive care unit for 18 hours. His postoperative course was uneventful. Ten days following his admission the patient was discharged in good condition, presenting however atrial fibrillation with good rate control under Amiodarone treatment. Three months following his discharge he remains free of symptoms.

## Discussion

The first case of SVCS was described by William Hunter in 1757 [1]. In the past benign pathology was the prime cause of this syndrome [2]. Currently this severe disease is caused mainly by tumors which compress or develop inside the superior vena cava. In recent reports, benign diseases accounted for 10% of SVCS [3]. Among these, dehiscence of anastomotic regions in previously operated patients for heart vessel reconstruction appears to be a very small group. However, in patients previously having such operations the possibility of a pseudoaneurysm should always be taken in consideration and guide towards a more detailed evaluation with enhance CT of the chest, independently of the presence of allergy history or renal dysfunction. Such a diagnostic approach may avoid unnecessary and risky attempts to biopsy a mediastinal mass or proceed with mediastinal irradiation which in the case of our patient, as well as in similar cases, would not resolve the superior vena cava syndrome.

Pseudoaneurysm of the ascending aorta is well known, however rare and potentially fatal complication after aortic surgery [4,5]. The sites of its origin are certain spots of handling during cardiac surgery such as the aortic cross clamp area, aortotomy, aortic cannulation and vent, cardioplegia needle, coronary anastomosis and proximal and distal aortic suture lines [6,7]. The mechanism of pseudoaneurysm formation is not yet clear, but suture line tension and persistent bleeding into the space between the graft and the wrapped aorta wall seem to be the most important [8]. Its frequency, higher in older series of patients [9-12], has been decreased by the avoidance of the inclusion and wrap techniques, the improvement of the graft's fabrication materials, the use of earlier re-exploration for bleeding and the improvement made in surgical experience [13]. In the report of 172 operated patients by Koutsoukos et al [6] the rate of pseudoaneurysm formation was 5.8% with only a single patient presenting such complication when the inclusion and wrap techniques were not used. More recently Mohammadi and colleagues [13] reported only 15 cases of ascending aorta pdeudoaneurysm among 885 patients undergoing surgery for ascending thoracic aorta aneurysm or aortic dissection.

In the presence of a pseudoaneurysm surgery is mandatory with intent to repair the aortic defect under conditions of safe re-sternotomy to avoid impeding rupture of the aneurysm and cerebral malperfusion. Institution of a femoro-femoral bypass, and proceeding with deep hypothermic circulatory arrest, prior to sternal re-entry, represents a safe technique to avoid intraoperative bleeding and exsanguination [12,14]. Nevertheless, other techniques such as partial circulatory arrest with antegrade perfusion of the carotid arteries and the establishment of cardiopulmonary bypass via axillary or femoral cannulation with concomitant use of remote access perfusion aortic cannula to avoid deep hypothermia, have also been reported [13,15,16]. Recently, endovascular approaches by means of stent grafts or septal occluders employ have been used to treat ascending aorta pseudoaneurysms [17,18]. Endovascular approaches may represent a valid alternative for the high operative risk patients; however they should not be used in the presence of mass effect symptoms, as in our patient, where the relief of the compression through the removal of the thrombotic material is mandatory.

## Conclusions

Dehiscence from the proximal or distal aorto-graft anastomosis can occur long after an aortic composite graft operation. It is a necessity to consider the formation of pseudoaneurysm as a rare but dramatic complication in almost all cardiovascular operations [12,19,20] and we should always have in mind the possibility of mass effects on adjacent mediastinal structures such as the superior vena cava. Enhanced CT of the chest should always be performed to confirm the syndrome's diagnosis and differentiate between a solid mass and a pseudoaneurysm in patients previously having heart surgery. Surgery should point to the repair of the defect with safe approaches to avoid bleeding and cerebral malperfusion.

## Competing interests

The authors declare that they have no competing interests.

## Consent

Written informed consent was obtained from the patient for publication of this case report and accompanying images. A copy of the written consent is available for review by the Editor-in-Chief of this journal.

## Authors' contributions

All authors have made substantial contributions to conception and design, or acquisition of data, or analysis and interpretation of data and have been involved in drafting the manuscript or revising it critically for important intellectual content. All authors read and approved the final manuscript.

PD: Manuscript Preparation, Literature Search, Study Design; IN: Manuscript Preparation, Literature Search; PH: Data Interpretation, Literature Search; CP: Manuscript Preparation, Literature Search; PD: Data Interpretation, Literature Search; EA: Data Interpretation, Literature Search; ENK: Manuscript Preparation, participated in its design and coordination.
